# The intricate interplay between cancer stem cells and cell-of-origin of cancer: implications for therapeutic strategies

**DOI:** 10.3389/fonc.2024.1404628

**Published:** 2024-05-10

**Authors:** Oluwaseun Adebayo Bamodu, Chen-Chih Chung, Thomas R. Pisanic, Alexander T. H. Wu

**Affiliations:** ^1^ Directorate of Postgraduate Studies, School of Clinical Medicine, Muhimbili University of Health and Allied Sciences, Dar es Salaam, Tanzania; ^2^ Ocean Road Cancer Institute, Dar es Salaam, Tanzania; ^3^ Department of Neurology, Taipei Medical University - Shuang Ho Hospital, New Taipei City, Taiwan; ^4^ Department of Neurology, School of Medicine, College of Medicine, Taipei Medical University, Taipei, Taiwan; ^5^ Taipei Neuroscience Institute, Taipei Medical University - Shuang Ho Hospital, New Taipei City, Taiwan; ^6^ Johns Hopkins Institute for NanoBioTechnology, Baltimore, MD, United States; ^7^ Sidney Kimmel Comprehensive Cancer Center, The Johns Hopkins University School of Medicine, Baltimore, MD, United States; ^8^ Department of Oncology - Cancer Genetics and Epigenetics, Johns Hopkins University, Baltimore, MD, United States; ^9^ The Program for Translational Medicine, Graduate Institute of Biomedical Informatics, College of Medical Science and Technology, Taipei Medical University, Taipei, Taiwan; ^10^ TMU Research Center of Cancer Translational Medicine, Taipei Medical University, Taipei, Taiwan; ^11^ Clinical Research Center, Taipei Medical University Hospital, Taipei Medical University, Taipei, Taiwan; ^12^ Graduate Institute of Medical Sciences, National Defense Medical Center, Taipei, Taiwan

**Keywords:** cancer stem cells, cell-of-origin, tumorigenesis, tumor heterogeneity, cancer therapeutics, therapy resistance, personalized medicine, tumor microenvironment

## Abstract

**Background:**

Cancer stem cells (CSCs) have emerged as pivotal players in tumorigenesis, disease progression, and resistance to therapies.

**Objective:**

This comprehensive review delves into the intricate relationship between CSCs and the cell-of-origin in diverse cancer types.

**Design:**

Comprehensive review of thematically-relevant literature.

**Methods:**

We explore the underlying molecular mechanisms that drive the conversion of normal cells into CSCs and the impact of the cell-of-origin on CSC properties, tumor initiation, and therapeutic responses. Moreover, we discuss potential therapeutic interventions targeting CSCs based on their distinct cell-of-origin characteristics.

**Results:**

Accruing evidence suggest that the cell-of-origin, the cell type from which the tumor originates, plays a crucial role in determining the properties of CSCs and their contribution to tumor heterogeneity.

**Conclusion:**

By providing critical insights into the complex interplay between CSCs and their cellular origins, this article aims to enhance our understanding of cancer biology and pave the way for more effective and personalized cancer treatments.

## Introduction

Cancer is a complex and multifactorial disease characterized by uncontrolled cell proliferation and invasion (1). The concept of cancer stem cells (CSCs) has revolutionized our understanding of tumor initiation, progression, and therapeutic resistance. CSCs possess self-renewal and differentiation capabilities, contributing to tumor heterogeneity and therapeutic resistance (2, 3). Recent studies suggest the cellular origin of cancer impacts CSC properties and behavior (4, 5). This review explores the relationship between CSCs and cell-of-origin across cancer types. We analyze mechanisms driving CSC formation, examine how cell-of-origin influences CSCs and tumor progression, discuss implications for cancer therapy, and suggest future research directions to enhance understanding of this complex interplay.

The notion of CSCs challenges the traditional view of cancer as a homogeneous mass of rapidly dividing cells, and instead, emphasizes the presence of a hierarchical organization within tumors (6). According to this model, CSCs serve as the “seeds” of the tumor, while the non-tumorigenic, differentiated cancer cells constitute the “bulk” or “bulk tumor” (7). This hierarchical organization implies that the eradication of CSCs is crucial for achieving long-term tumor remission and preventing relapse, as CSCs have the capacity to regenerate and drive tumor regrowth even after initial therapy-induced tumor regression (8).

Notably, recent research has illuminated a fascinating and nuanced relationship between the cellular origin of cancer and the properties of CSCs. The cell-of-origin refers to the normal cell type that accumulates the initiating mutations and transforms into a cancerous cell (9). Evidence suggests that the cell-of-origin exerts a profound influence on CSC behavior, dictating their characteristics, tumorigenic potential, and response to therapeutic interventions (10, 11).

In conclusion, the relationship between CSCs and the cell-of-origin represents a compelling and intricate area of cancer research. Understanding the impact of the cellular origin of cancer on CSC phenotypes has the potential to unlock novel therapeutic avenues and advance precision medicine in oncology. By elucidating the molecular and cellular underpinnings of this relationship, we aim to contribute significantly to the growing body of knowledge in cancer biology and ultimately pave the way for more effective and personalized cancer treatments.

## Cancer stem cells origin and properties

CSCs, a small subset of tumor cells possessing stem cell properties, including self-renewal and multipotent differentiation capacity, are the root cause of tumor initiation, therapeutic resistance, metastasis, and relapse (12, 13). Though functionally defined by their tumor-propagating ability, their cell-of-origin and relationship to normal stem cells is debated (14).

CSCs were initially thought to arise from normal tissue stem cells that accumulate mutations enabling aberrant self-renewal (15). Evidence now suggests that more committed progenitors or differentiated cells may acquire self-renewal capacity through dysregulation of embryonic stem cell programs (14). Regardless of origin, CSCs are defined by expression of stem cell markers and pathways regulating self-renewal and survival (7). Well-studied CSC markers include CD44, CD133, and aldehyde dehydrogenase (ALDH), but their specificity is context-dependent (4). Intrinsic and microenvironmental factors all contribute to generate CSC populations with heterogeneous phenotypes and plasticity (16).

CSCs’ increased tumorigenicity upon limiting dilution xenotransplantation reflects their self-renewal capacity (17). CSCs propagate tumors long-term and recapitulate intratumor heterogeneity, differentiating into non-CSC bulk tumor cells (18). Beyond initiating tumor growth, CSCs mediate metastasis and therapeutic resistance through quiescence, enhanced DNA repair, drug efflux pumps, anti-apoptotic signaling, and immunosuppressive effects (19).

However, CSCs exhibit plasticity, readily interconverting between stem and non-stem states (20). The variability in intratumoral proportion of CSCs, coupled with the non-CSCs’ capacity to dedifferentiate into CSCs, especially post-therapy, questions proposed CSC rarity (11, 16). This plasticity enables dynamic maintenance of CSC populations, challenging efforts to definitively isolate stable CSC subsets.

The presence of CSCs within tumors has critical implications for cancer therapy. Conventional cytotoxic treatments, such as chemotherapy and radiation, target rapidly dividing cells, which primarily constitute the non-CSC tumor bulk population, leading to initial shrinkage of tumor size, and often temporary tumor regression (21). However, CSCs upregulate pro-survival signaling pathways and overexpress ATP-binding cassette (ABC) drug efflux transporters, conferring resistance to chemotherapeutics, survival advantage, metastasis, and recurrence (4, 22, 23). These intrinsic resistance mechanisms allow CSCs to evade conventional treatments, survive initial therapy and eventually repopulate the tumor, leading to recurrence and metastasis (5). For instance, glioblastoma CSCs expressing Hedgehog, Notch, and angiogenic pathways were found to persist after temozolomide chemotherapy, reconstituting recurrent tumors (24). Therefore, understanding the properties and behaviors of CSCs is vital for developing tailored strategies, like combination therapies or inhibitors targeting specific resistance pathways, to eradicate these resilient cell populations and improve long-term treatment outcomes (25).

## Cell-of-origin and its influence on cancer stem cells

The cell-of-origin concept proposes that stemness properties are largely shaped by the normal cell type from which CSCs arise (11). Extensive evidence now indicate that the specific cellular context in which cancer-initiating mutations occur influences downstream CSC behavior, including tumorigenicity, differentiation capacity, and therapeutic vulnerability (4, 14).

Studies across cancer types show the relationship between cell-of-origin and CSC properties. In glioblastoma, neural stem cell-derived CSCs propagated more infiltrative, aggressive tumors compared to CSCs from committed neural progenitors (24). In prostate cancer, basal cell-derived CSCs generated tumors with higher Gleason scores and metastases than luminal cell-derived CSCs (26), and in colorectal cancer, studies have demonstrated that colorectal CSCs derived from intestinal stem cells exhibit a higher tumorigenic potential and metastatic ability compared to those originating from more differentiated cell types. Specifically, Lgr5+ intestinal stem cell-derived CSCs preferentially metastasized to the liver and express liver homing chemokine receptors, such as CCR6 (27).

In pancreatic cancer, the cell-of-origin plays a crucial role in determining the characteristics of pancreatic CSCs (PCSCs). PCSCs derived from pancreatic progenitor cells display enhanced self-renewal and tumorigenic potential compared to those originating from differentiated acinar or ductal cells (28, 29). Additionally, the cell-of-origin influences the activation of specific oncogenic signaling pathways in PCSCs, with progenitor-derived PCSCs exhibiting aberrant Hedgehog pathway activation (30).

The cell-of-origin has been implicated in shaping the behavior of lung CSCs (LCSCs). LCSCs derived from basal stem cells in the airway epithelium exhibit increased invasiveness and metastatic potential compared to those originating from other cell types (31, 32). Furthermore, the cell-of-origin determines the expression of specific stem cell markers and the activation of distinct signaling pathways in LCSCs, influencing their response to targeted therapies (30, 32).

More so, mesenchymal stem cells (MSCs) have emerged as potential cells-of-origin for CSCs in various cancer types, including glioblastoma. Recent studies have suggested that MSCs may contribute to the formation and maintenance of CSCs through various mechanisms - In glioblastoma, MSCs have been implicated as potential cell-of-origin for a CSC subset exhibiting mesenchymal phenotype (33–35). These mesenchymal glioblastoma stem cells, akin to their neural stem cell-derived CSCs counterparts, are associated with increased invasiveness, resistance to therapy, and poor prognosis (35). Emerging evidence suggests that the transformation of MSCs may give rise to this aggressive glioblastoma subpopulation (34, 35).

Aside glioblastoma, MSCs have been proposed as potential cells-of-origin for CSCs in various other cancer types, including breast cancer (36, 37), prostate cancer (38), and osteosarcoma (37, 39). The capacity of MSCs to differentiate into multiple lineages and their inherent migratory and self-renewal properties may contribute to their potential role in CSC formation and tumor progression (33, 34, 36, 37). The role of MSCs as cells-of-origin for CSCs is an active area of research, and further investigation is needed to elucidate the mechanisms underlying this potential relationship and its implications for cancer development and therapeutic strategies.

Mechanistically, the cell-of-origin imprints durable epigenetic, transcriptional, and signaling programs that shape CSC behavior (40). The cell-of-origin dictates activation of distinct oncogenic pathways, as basal breast CSCs upregulate EGFR while luminal CSCs activate HER2 signaling (41). Additionally, the cell-of-origin determines CSC differentiation trajectories, with mature cells generating unipotent CSCs while early progenitors produce multipotent CSCs (10).

Importantly, the cell-of-origin also modulates therapeutic response. Breast CSCs from basal cells resist radiation but remain DNA damage-sensitive, whereas those from luminal cells upregulate ABC drug transporters (42). In melanoma, CSCs derived from blocked differentiated cells retain DNA damage response mechanisms and are readily targetable, compared to those from neural crest stem cells (43). Thus, personalized, context-specific anti-CSC therapies are possible, but challenged by intratumor heterogeneity and CSC plasticity. Integrating lineage tracing, single-cell profiling, and functional validation is critical for understanding these pivotal interactions.

## Cell-of-origin based molecular mechanisms driving cancer stem cell formation

The cell-of-origin influences the molecular events that enable normal cells transform into CSCs. This cellular context determines the signaling pathways, epigenetic programs, and mutations that confer aberrant self-renewal ability. Cells-of-origin shape mutations arising during CSC formation. Different cells-of-origin possess distinct DNA repair deficiencies that allow specific mutations. For example, melanocyte stem cells have high levels of reactive oxygen species and rely heavily on nucleotide excision repair (NER) (44). Mutations in NER genes like ERCC2 in melanocyte stems cells allow DNA damage accumulation, hypermutability, and formation of melanoma CSCs (45). In contrast, mammary stem cells are deficient in homologous recombination (HR) repair due to epigenetic repression of BRCA1 (46). Hence, loss of BRCA1/2 occurs early during breast CSC formation, enabling genomic instability through HR deficiency (47).

Several key signaling pathways involved in normal stem cell biology become dysregulated in CSCs in a cell-of-origin dependent manner. For instance, Wnt pathway mutations in intestinal stem cells promote colorectal CSC formation, while Hedgehog activation drives CSC properties in Sonic Hedgehog-responsive cerebellar stem cells giving rise to medulloblastoma (48, 49). The cell-of-origin also determines which signaling pathways are leveraged for CSC formation. For example, normal hematopoietic stem cells require FGF signaling but rely on BMP signaling for differentiation (50). Mutations activating FGF signaling while inhibiting BMP signaling promote aberrant self-renewal, allowing hematopoietic stem cells to transform into leukemic stem cells (50). A similar scenario occurs in glioblastoma, where mutations activating growth factor signaling like EGFR/PDGFRα in neural stem cells drive unrestrained proliferation during CSC genesis (51).

The epigenetic and metabolic states established by the cell-of-origin play important roles in CSC reprogramming. Normal intestinal stem cells exhibit an “open” chromatin landscape at Wnt target gene loci which primes them for CSC formation when APC mutations occur, enabling aberrant TCF/β-catenin transcriptional activation of stemness signals (52, 53). Similarly, mammary stem cells possess an epigenetic landscape suppressing BRCA1 but permitting proliferation, which fosters CSC properties when BRCA1 is mutated (46). Moreover, normal intestinal stem cells rely heavily on oxidative phosphorylation, which is co-opted during colon CSC formation to sustain stemness, and mediated in part by mutations in fumarate hydratase (54, 55).

Appreciating how the cell-of-origin-dependent genomic, epigenetic, and metabolic states shape the specific mechanisms enabling CSC formation is crucial for developing novel approaches to prevent oncogenic transformation by targeting these early vulnerabilities in a cell-context-specific manner.

## Microenvironmental regulation of cancer stem cell maintenance and survival

**Figure 1:**
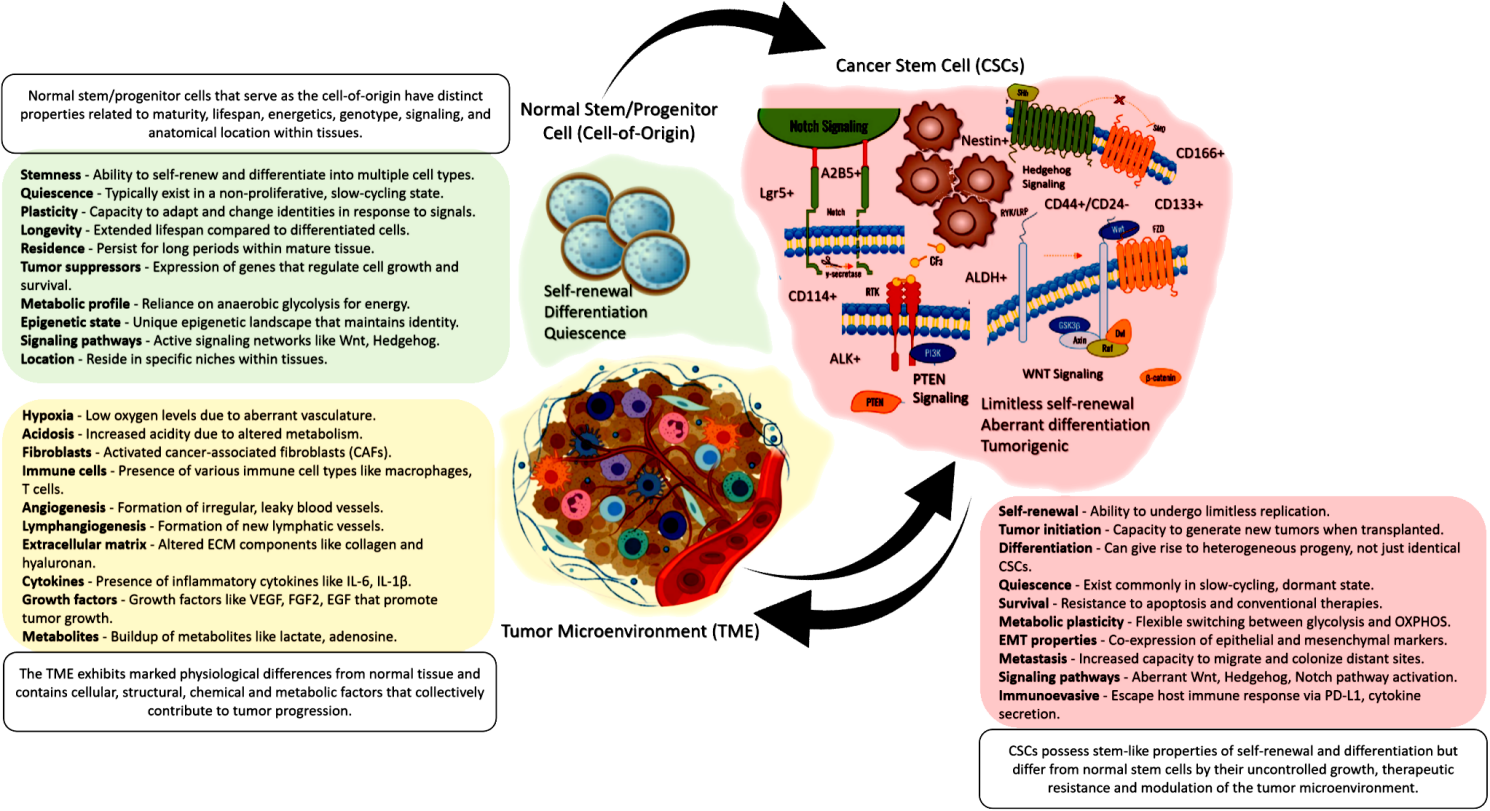
A visual summary showing that normal tissue stem/progenitor cells give rise to CSCs, which then interact with and reshape the surrounding tumor microenvironment. This illustrates the critical interplay between cell-of-origin, CSCs, and the TME in cancer.

The tumor microenvironment (TME) is now recognized as a critical regulator of CSC fate and function (**Figure 1**). Rather than acting in isolation, CSCs engage in dynamic crosstalk with surrounding stromal, immune, endothelial, and ECM components that maintain stemness properties and confer therapeutic resilience (56). Elucidating these complex TME interactions offers exciting opportunities for new anti-CSC therapies.

### Hypoxia

Hypoxia, a characteristic feature of the tumor microenvironment (TME) which is almost always coupled with chaotic vasculature and rapid cell proliferation, has emerged as a critical regulator of CSC maintenance and survival. Accruing evidence implicate hypoxia in the upregulation of multi-drug resistance transporters, maintenance of undifferentiated state, and enhancement of tumorigenic potential of CSCs (57, 58). Low oxygen tension stabilizes hypoxia-inducible factors (HIFs), which transcriptionally activate genes involved in stemness, self-renewal, and therapy resistance (59–62). Specifically, HIF-1α and HIF-2α promote the expression of pluripotency factors like Oct4, Nanog, and Sox2, as well as the upregulation of ABC drug efflux transporters, contributing to the chemoresistance of CSCs (59, 60). Additionally, hypoxia induces the expression of CSC markers, such as CD44 and CD133, and enhances the sphere-forming ability of CSCs, indicative of their self-renewal capacity (13, 61, 62). Targeting hypoxia-activated pathways thus represents a promising approach to disrupt the CSC-promoting effects of hypoxic TMEs.

### Inflammation

Beyond hypoxia, the TME is often characterized by chronic inflammation, which plays a crucial role in shaping the CSC phenotype. TME-derived inflammatory signals expand CSCs through cytokine-mediated activation of NF-κB, STAT3, and other critical stemness pathways (63). Inflammatory cytokines, such as IL-6, IL-8, and TNF-α, secreted by tumor-associated immune cells and stromal components, activate pro-survival signaling pathways like NF-κB, STAT3, and Akt in CSCs (63, 64). These pathways upregulate stemness-associated transcription factors, including Oct4, Nanog, and c-Myc, promoting self-renewal and therapy resistance (64, 65). Furthermore, inflammation-induced EMT has been linked to the acquisition of stem-like properties and the generation of CSCs from non-stem tumor cells (66, 67). It is also notable that checkpoint blockade elicits T cell production of inflammatory cytokines that may inadvertently expand CSCs by stimulating these pathways (63).

### Stromal cell interactions

The dynamic crosstalk between CSCs and non-malignant stromal components of the TME, such as cancer-associated fibroblasts (CAFs), endothelial cells, and mesenchymal stem cells (MSCs), plays a pivotal role in regulating CSC behavior. Paracrine signaling networks involving cytokines, growth factors, and metabolites facilitate bidirectional communication between CSCs and stromal cells (68–70). For instance, CAF-derived factors like TGF-β, CXCL12, and IL-6 induce EMT, stemness, and therapy resistance in CSCs, while CSC-secreted factors like IL-1β and IL-8 promote a pro-tumorigenic phenotype of CAFs, including EMT, drug resistance, and metastasis (71–74). Similarly, endothelial cell-derived factors stimulate CSC self-renewal and angiogenesis, while MSCs contribute to the maintenance of CSC populations through cytokine signaling and cell-cell interactions (71, 74). Targeting the homeostatic mechanisms by which non-malignant stroma supports CSC maintenance may offer new therapeutic inroads.

### Extracellular matrix remodeling

The extracellular matrix (ECM) cues within the TME provides crucial biochemical and biomechanical cues that regulate CSC behavior and fate. The composition, organization, and stiffness of the ECM influence CSC self-renewal, division patterns, differentiation, plasticity, and therapy resistance (75, 76). Adhesive ECM proteins like laminin induce symmetric division that propagates the CSC pool, whereas fibrillar collagen I promotes differentiation (77). Specific ECM components, such as laminin and hyaluronic acid, have been shown to promote CSC self-renewal and stemness, while others, like collagen I, induce differentiation (75–77). Additionally, matrix metalloproteinases (MMPs) secreted by CSCs and stromal cells remodel the ECM, releasing bioactive fragments that modulate CSC signaling and promote invasion and metastasis (76, 78, 79). ECM stiffness has also been implicated in regulating CSC plasticity, with increased matrix rigidity favoring the acquisition of a mesenchymal-like CSC phenotype associated with enhanced invasiveness and therapy resistance (78, 79). Manipulating ECM signals to silence self-renewal and promote differentiation offers a promising approach to deplete CSCs.

## Cell-of-origin dynamics shaping cancer stem cell-driven tumorigenesis, invasion, and metastasis

The cell-of-origin is a critical determinant of cancer pathogenesis, shaping tumor initiation, growth rate, and metastatic potential. Comparative studies exploring different cell-of-origin scenarios within specific cancers have shed light on the complexity of tumor heterogeneity and have implications for personalized cancer therapies.

### Tumor initiation

Different cell types possess distinct genetic and epigenetic susceptibilities, making some more prone to oncogenic transformation (3). In glioblastoma, CSCs derived from neural stem cells generate more rapidly expanding and invasive tumors than CSCs from progenitor cells, reflecting greater self-renewal and motility (24). This indicates cells-of-origin dictate the degree of stemness and aggressiveness of resultant CSC populations.

Moreover, certain cell-of-origin contexts favor more aggressive tumor initiation. For example, basal cell-derived prostate cancer is more aggressive and associated with higher Gleason scores than luminal cell-derived cancers (80). Similarly, in breast cancer, mammary stem cells have been identified as a cell-of-origin that gives rise to tumors with a basal-like phenotype, which typically carries a poor prognosis (9). These findings underscore the significant influence of cellular context on the initial transformation events that set the stage for tumor development.

### Tumor heterogeneity

The cell-of-origin controls CSC differentiation trajectories, thus, influencing downstream heterogeneity. For instance, mature luminal cells transformed into prostate CSCs produce unipotent tumors composed predominantly of luminal cells (26). However, multipotent basal stem cells yield heterogeneous prostate cancers encompassing luminal, basal, and neuroendocrine cells *via* their multipotent CSC derivatives (26).

### Tumor growth rate

The cell-of-origin can also impact the growth rate of tumors. CSCs derived from highly proliferative and undifferentiated progenitor cells may contribute to more rapidly growing tumors (11), whereas, those originating from more differentiated cell types with limited self-renewal capacity may exhibit slower growth rates (5). The cellular background can imprint specific molecular signatures on CSCs, influencing their proliferative capacity and tumor growth dynamics (7).

Moreover, the cell-of-origin can influence the overall aggressiveness and invasive behavior of the tumor. In glioblastoma, for instance, tumors arising from neural stem cells tend to exhibit more extensive infiltration into surrounding brain tissues compared to those originating from neural progenitors (81). These distinctions in growth rate and invasiveness emphasize the importance of cell-of-origin in understanding tumor behavior and designing effective therapeutic strategies.

### Metastatic potential

The cell-of-origin shapes organotropism and patterns of metastasis, by influencing migratory pathways activated in CSCs. Intestinal stem cell-derived colorectal CSCs preferentially metastasize to liver due to expression of liver homing chemokine receptors like CCR6 (27). Gastric stem cell-derived CSCs favor distant metastasis through activation of a gastric carcinoma related protein CARP-1 (82). Similarly, luminal breast cancers have been shown to preferentially metastasize to bone, while basal-like breast cancers tend to metastasize to the brain and lungs (83). In essence, the cell-of-origin has profound impacts on subsequent tumor evolution and progression kinetics. This knowledge promises to elucidate the molecular determinants of tumorigenesis and reveal actionable differences between cancers arising from distinct cells-of-origin.

### Epithelial-to-mesenchymal transition

The epithelial-to-mesenchymal transition (EMT) is a fundamental process in embryonic development and tissue remodeling, wherein epithelial cells lose their polarity and cell-cell adhesion properties, acquiring a mesenchymal phenotype with increased migratory and invasive capabilities (66, 67, 72, 78). Emerging evidence suggests that EMT plays a crucial role in the generation and maintenance of CSCs, and this process is closely linked to the cell-of-origin. In epithelial cancers, such as breast and prostate cancer, EMT has been implicated in the formation of CSCs from more differentiated epithelial cells (84, 85). During EMT, epithelial tumor cells acquire stem-like properties, including self-renewal, increased expression of CSC markers (e.g., CD44, CD24, and ALDH), and enhanced resistance to therapies (66, 67, 72, 78, 84, 85). The induction of EMT in these cells is often mediated by transcription factors like Twist, Snail, Slug, and Zeb1, which suppress epithelial markers like E-cadherin and upregulate mesenchymal markers like vimentin and N-cadherin (85–87).

Importantly, the propensity for EMT and the subsequent generation of CSCs is influenced by the cell-of-origin. In breast cancer, basal-like or triple-negative breast cancer cells, which are thought to originate from more primitive mammary stem/progenitor cells, exhibit a higher propensity for EMT and CSC formation compared to luminal subtypes derived from more differentiated epithelial cells (88). Similarly, in prostate cancer, basal cells are more prone to undergoing EMT and acquiring stem-like properties compared to luminal cells (89, 90). EMT not only contributes to the formation of CSCs but also plays a critical role in metastasis, another key feature of CSCs. EMT enables CSCs to disseminate from the primary tumor site, invade surrounding tissues, and eventually colonize distant organs, establishing metastatic lesions (89, 91). The interplay between EMT and CSCs is bidirectional, as CSCs can also induce EMT in neighboring tumor cells, further promoting metastasis and therapy resistance (89, 90, 92, 93).

### Metastatic cascades and organotropism

CSCs are pivotal drivers of metastatic dissemination and the establishment of secondary tumors in specific distant organs, a process known as organotropism. CSCs possess several characteristics that facilitate their ability to initiate and sustain metastatic cascades. CSCs exhibit enhanced invasive and migratory capabilities, enabling them to detach from the primary tumor, invade the surrounding stroma, and intravasate into the circulatory or lymphatic systems (89–93). This invasive behavior is mediated by the activation of EMT programs, as discussed earlier, and the expression of specific cell surface markers and proteases that facilitate extracellular matrix degradation and cell motility (72, 78, 85–87). Once in the circulation, CSCs possess intrinsic mechanisms that allow them to survive and evade immune surveillance. These include enhanced expression of anti-apoptotic proteins, increased DNA repair capacity, and the ability to enter a quiescent or dormant state (94, 95). This dormancy enables CSCs to withstand the harsh conditions of the circulatory system and later reactivate their proliferative and self-renewal capabilities upon reaching a suitable microenvironment. Upon extravasation into distant organs, CSCs demonstrate remarkable adaptability to the new microenvironment. They secrete factors that remodel the local niche, promoting angiogenesis, immune evasion, and the recruitment of supportive stromal cells (94, 95). This niche formation creates a permissive environment for CSC self-renewal, proliferation, and eventual establishment of metastatic lesions.

The preferential dissemination of CSCs to specific organs is known as organotropism, and it is influenced by the interplay between CSC-intrinsic factors and the unique microenvironmental cues of distant organs (96, 97). For example, in gallbladder tumors, CSCs expressing high levels of CXCR4 preferentially metastasize to the liver, where its ligand CXCL12 is abundantly expressed (98). Similarly, in pancreatic cancer, CSCs expressing the TEK receptor tyrosine kinase and integrin α6β4 exhibit a propensity for liver and lung metastasis, respectively (99, 100). In prostate cancer, CSCs expressing the bone-specific chemokine receptor CCR3 preferentially establish bone metastases (101). Lung metastasis in various cancers, including breast and colon cancer, has been linked to the expression of specific adhesion molecules and chemokine receptors on CSCs that facilitate their homing to the lung microenvironment (102, 103).

Understanding the intricate mechanisms underlying CSC-driven metastasis and organotropism, will help facilitate identification of potential therapeutic targets and develop strategies to disrupt these processes, ultimately improving patient outcomes.

## Epigenetic mechanisms in cancer stem cells: influence of the cell-of-origin

Epigenetic modifications, including DNA methylation, histone modifications, and non-coding RNA regulation, play crucial roles in shaping the behavior and properties of CSCs (104). Importantly, the epigenetic landscape of the cell-of-origin can have a profound impact on the epigenetic patterns observed in CSCs, influencing their stemness, self-renewal, and tumorigenic potential.

### DNA methylation patterns and CSC stemness

DNA methylation, the addition of methyl groups to cytosine residues in CpG dinucleotides, is a key epigenetic mechanism that regulates gene expression. Aberrant DNA methylation patterns, including hypermethylation of tumor suppressor genes and hypomethylation of oncogenes, are hallmarks of cancer and contribute to the acquisition of stem-like properties in CSCs (105–107).

Importantly, the cell-of-origin can impart specific DNA methylation patterns that shape the behavior of CSCs. For instance, in colorectal cancer, intestinal stem cells exhibit a distinct DNA methylation landscape primed for the activation of Wnt signaling, a critical pathway for stem cell self-renewal (108). When these cells acquire mutations in genes like APC, the pre-existing methylation patterns facilitate the aberrant activation of Wnt signaling, driving the formation of colorectal CSCs (53, 108, 109).

Similarly, in breast cancer, mammary stem cells possess an epigenetic landscape that represses the expression of the DNA repair gene BRCA1 (46). Mutations in BRCA1 in this context lead to genomic instability and the acquisition of stem-like properties, contributing to the formation of breast CSCs (46, 47). These examples illustrate how the cell-of-origin’s epigenetic landscape can predispose certain cell types to CSC formation upon accumulation of specific genetic alterations.

### Histone modifications and gene regulation in CSCs

Histone modifications, such as acetylation, methylation, phosphorylation, and ubiquitination, regulate chromatin structure and gene expression patterns. These modifications can influence the stemness and self-renewal properties of CSCs, and their dysregulation has been implicated in various cancers (110–112).

The cell-of-origin can contribute to the histone modification patterns observed in CSCs. For example, in glioblastoma, the histone methyltransferase G9a is markedly depleted in CD133+ neural stem cells, the proposed cell-of-origin for glioblastoma CSCs (113). Upregulated G9a expression induces histone H3 lysine 9 methylation (H3K9me2), which in turn downregulates the expression of stemness-associated genes. Conversely, aberrant G9a activity in NSCLC CSCs leads to increased stemness and tumorigenicity, potentially influenced by the epigenetic landscape inherited from the lung epithelial cells-of-origin, also known as tumor-initiating cells (107). In addition, in NSCLC, histone ubiquitination patterns have been linked to the regulation of CSC properties. The histone E3 ubiquitin ligase TRIM37 is highly expressed in NSCLC CSCs and promotes stemness and metastasis through the ubiquitination of histone H2A (114), suggesting a potential link between the epigenetic landscape of the cell-of-origin and the histone modification patterns observed in CSCs.

### Non-coding RNAs and CSC regulation

Non-coding RNAs, particularly microRNAs (miRNAs) and long non-coding RNAs (lncRNAs), have emerged as critical regulators of CSC properties and behavior. These non-coding RNAs can modulate gene expression by targeting mRNAs for degradation or translational repression, or by influencing epigenetic mechanisms (106, 110).

The expression and function of non-coding RNAs in CSCs are cell-of-origin-dependent. For instance, in glioblastoma, the lncRNA NEAT1 is highly expressed in neural stem cells and contributes to the maintenance of stemness and self-renewal (115). Conversely, NEAT1 has been shown to suppress stem cell self-renewal and leukemogenesis by inactivating Wnt signaling (116), consistent with its role as a tumor suppressor, and as a direct transcriptional target of the tumor suppressor gene p53 (117). Furthermore, NEAT1 has been found to mitigate multidrug resistance in leukemia by inhibiting the ABCG2 gene (118).

The long non-coding RNA MIR22HG is also upregulated in glioblastoma CSCs, where it promotes stemness and therapy resistance by regulating epigenetic changes and gene expression programs (119). However, MIR22HG plays tumor-suppressive role in various other types of cancer, including lung cancer, hepatocellular carcinoma, endometrial cancer, gastric cancer, and cholangiocarcinoma (120–122). Its low expression is associated with poor prognosis in these cancers. MIR22HG exerts its tumor-suppressive effects through various mechanisms, including the suppression of proliferation, invasion, and metastasis, and the attenuation of CSC-activating NOTCH2 signaling (120–123). This indicates that the cell-of-origin’s lncRNA expression patterns is retained in CSCs and contribute to their stemness properties.

Moreover, in breast cancer, the miRNA expression profiles of CSCs have been shown to be influenced by the cell-of-origin. Basal-like breast CSCs derived from more primitive mammary stem/progenitor cells exhibit distinct miRNA signatures compared to luminal breast CSCs derived from more differentiated cells (124, 125). These miRNA profiles can regulate key stemness-associated pathways and contribute to the differences in CSC properties observed between different breast cancer subtypes.

Increased understanding of the interplay between epigenetic mechanisms and the cell-of-origin in shaping CSC behavior will aid identification of potential therapeutic targets and develop epigenetic-based strategies to disrupt CSC maintenance and self-renewal, potentially leading to more effective cancer treatments.

## Leveraging the integrative understanding of cancer stem cell biology and cell-of-origin effects in cancer therapies

Deciphering the intricate relationship between CSCs and their cell-of-origin provides unprecedented opportunities to transform cancer therapies through personalized, mechanism-based approaches. However, to effectively leverage these paradigm-shifting discoveries to conquer tumor heterogeneity, evolution, and resistance necessitates deliberate integration of this knowledge into complex therapeutic designs rooted in a deep appreciation of cancer biology.

Cell-of-origin insights can guide predictive biomarker-driven patient stratification to optimize chemotherapy regimens. For instance, gene expression profiling of breast CSCs revealed distinct chemotherapy vulnerability profiles based on the cell-of-origin-linked intrinsic subtype, which facilitates predictive selection of taxane *versus* anthracycline-based regimens to improve outcomes (126, 127). Furthermore, rational co-targeting of activated oncogenic drivers in CSCs through combinatorial chemotherapy regimens, as demonstrated by EGFR blockade enhancing taxane therapy in basal breast cancers, disables key survival pathways perpetuating drug resistance (128). However, conquering acquired resistance requires accounting for CSC plasticity and inevitable clonal selection. Innovative adaptive designs that dynamically adjust dosing in response to altered CSC composition show promise in this context (129).

Regarding radiotherapy, the radiosensitivity phenotype of CSCs strongly associates with cell-of-origin determinants. Compared to CSCs arising from differentiated cells that retain residual DNA repair capacity, those originating from undifferentiated stem/progenitor cells upregulate robust pro-survival signaling and display relative radioresistance (24). Targeted radiosensitization through inhibitors disabling these radioprotective CSC programs, informed by cell-of-origin insights, as with Chk1 inhibitors in laryngeal and tongue CSCs, can dramatically improve therapeutic index (130). However, the sequencing and schedule of radiotherapy combinations warrants careful optimization to avoid potential expansion of aggressive CSC subclones.

Moreover, integrative genomic and single-cell profiling can help identify cell-of-origin-associated neoantigens for personalized vaccines or CAR-T cells targeting CSCs, as demonstrated against EGFRvIII-expressing glioblastoma CSCs of neural stem origin (131, 132). However, mitigating the immunosuppression conferred by the protective CSC niche remains crucial, highlighting opportunities for immunomodulators blocking inhibitory ligands like PD-L1 in CSCs (133). Cell-of-origin insights also empower selection of targeted therapies, such as KRAS wild-type status in intestinal crypt-derived colon CSCs, predicting sensitivity to EGFR blockade (53). Nevertheless, acquired resistance is inevitable due to subclonal evolution; single-cell genomic monitoring of CSC dynamics during therapy facilitates real-time adjustment of targeted regimens to overcome resistance emergence (134).

Further, cell-of-origin-associated CSC biomarker panels can enhance early detection and therapeutic monitoring. Longitudinal tracking of circulating CSCs expressing normal mammary stem cell markers may signal occult metastases, while shifting to mesenchymal markers can indicate early treatment failure (135). Liquid biopsy assessing CSC dynamics thereby enables personalized surveillance strategies.

## Therapeutic targeting of cancer stem cells based on cell-of-origin vulnerabilities

The cell-of-origin concept provides a framework for developing more precise therapies tailored to the unique vulnerabilities of CSCs based on their cellular origin.

### Small molecule inhibitors

Small molecule inhibitors designed against oncogenic drivers and signaling pathways activated preferentially in CSCs based on cell-of-origin are a promising therapeutic approach. In basal breast cancers enriched in mammary stem cell-derived CSCs, EGFR tyrosine kinase inhibitors like neratinib suppress pro-survival signaling in the CSCs (136). Wnt-targeting porcupine inhibitors, including LGK974, selectively inhibit Wnt-dependent intestinal stem cell-derived colorectal CSCs (137). Sonic Hedgehog (SHH) inhibitors vismodegib and glasdegib demonstrate activity against cerebellar stem cell-derived medulloblastoma CSCs exhibiting aberrant SHH pathway activation (138). Ongoing research aim to expand the repertoire of targeted CSC inhibitors informed by cell-of-origin (see **Tables 1**, **2**).

**Table 1:** Novel preclinical therapies targeting CSCs based on cell-of-origin to resolve chemotherapy resistance in solid tumors.

Cancer Type	CSC Markers	Therapies	Route	Side Effects	Models	References
Breast Cancer	ALDH+, CD44+/CD24-	Disulfiram	Oral	Nausea, neuropathy	PDX	(139)
		Oncolytic virus MG1MAGEA3	Intratumoral injection	Fever, fatigue	PDX	(140)
Colorectal Cancer	Lgr5+, CD44+, CD166+	LGK974	Oral	Well tolerated	PDX	(141)
		Napabucasin	Oral	GI toxicity	PDX	(142)
Glioblastoma	CD133+, Nestin+, A2B5+	WP1066	Intraperitoneal	No data	Intracranial xenografts	(143)
		GDC-0449	Oral	Muscle spasms	Intracranial xenografts	(144)
Cervical Cancer	CD44+, CD133+	Salinomycin	Intraperitoneal	No data	Xenografts	(145)
Ovarian Cancer	ALDH1+, CD117+	Disulfiram	Oral	Nausea, neuropathy	Xenografts	(146)
		ATRA	Oral	Headache, dry skin	Xenografts	(147)
Pancreatic Cancer	CD44+/CD24+/ESA+	LDE225	Oral	Well tolerated	PDX	(148, 149)
Neuroblastoma	CD114+, ALK+	ALK inhibitors (Crizotinib)	Oral	Vision changes, edema	Xenografts	(150)
		mTOR inhibitors (Rapamycin, Everolimus)	Oral	Stomatitis, rash	Xenografts	(151)
Bladder Cancer	CD44+, 67LR+, ALDH1A1+	EF24	Intravesical	No data	Xenografts	(152, 153)
Prostate Cancer	CD44+/α2β1+/ALDH1A1+	Niclosamide	Oral	GI toxicity	Xenografts	(154)
Kidney Cancer	CD105+, CD133+	mTOR inhibitor (WYE-687, CC-115)	Oral	Stomatitis, hyperglycemia	PDX	(155–157)
NSCLC	ALDH+, CD133+, MRP1+	Disulfiram	Oral	Nausea, neuropathy	PDX	(139)
		Oncolytic virus ZD55-TRAIL	Intratumoral injection	Fever, fatigue	Orthotopic models	(158)
Lung Cancer	APN/CD13+	Aminopeptidase inhibitor (Tosedostat)	Oral	No data	Xenografts	(159)
		ATRA	Oral	Headache, dry skin	Xenografts	(160)
Hepatocellular Carcinoma	CD90+, CD44+, EpCAM	GSK126	Intraperitoneal	No data	Xenografts	(161)

NSCLC, non-small cell lung cancer; ATRA, all-trans retinoic acid; PDX, patient-derived xenografts.

**Table 2:** Key clinical trial-based therapeutic advances in targeting cancer stem cells based on cell-of-origin between 2015 and 2023.

Cancer Type	Cell-of-Origin	CSC Markers	Therapeutic Advances	Mechanism	Clinical Trials	References
Breast Cancer	Luminal progenitor cells	CD44+, CD24-, ALDH+	Anti-CD44 antibody drug conjugates with Trastuzumab emtansine (T-DM1)	Targets CSC surface markers	Phase I trial of anti-CD44-T-DM1	(162, 163)
Glioblastoma	Neural stem cells	CD133+, Nestin+	Oncolytic virus therapy	Targets CSC self-renewal pathways	Phase I trial of oncolytic adenovirus Delta-24-RGD	(164, 165)
Colorectal Cancer	Intestinal stem cells	Lgr5+, CD44+	Wnt signaling inhibitor (Porcupine inhibitor CGX1321)	Blocks CSC maintenance pathways	Phase I and 1b trials of porcupine inhibitor CGX1321	(166)
Cervical Cancer	Cervical epithelial stem cells	CD44+, ALDH1+	Histone deacetylase inhibitors	Epigenetic modulation of CSCs	Phase I trial of PCI-24781 and MS-275 (Entinostat)	(167)
Ovarian Cancer	Ovarian epithelial stem cells	CD133+, CD117+	Anti-CD133 CAR T-cell therapy	Targets CSC antigen	Phase I trial of CD133 CAR T cells	(168)
Lung Cancer	Bronchioalveolar stem cells	CD166+, EpCAM+	DNA methyltransferase inhibitor (Gemcitabine)	Epigenetic regulation of CSCs	Phase I/II trials of gemcitabine	(169, 170)
Hepatocellular Carcinoma	Hepatic progenitor cells	CD90+, CD133+, EpCAM+, ALDH+	Sorafenib	Inhibits CSC signaling pathways	Approved for advanced HCC in 2007	(171, 172)
Pancreatic Cancer	Pancreatic epithelial progenitor cells	CD133+, CD44+	Metformin	Targets CSC metabolism	Phase I/II trials	(169 173, 174)
Neuroblastoma	Neural crest stem cells	CD114+, CD56+	Anti-GD2 immunotherapy	Targets CSC surface antigen	Phase III trials of dinutuximab	(175, 176)
Bladder Cancer	Bladder epithelial stem cells	CD44v6+, CK14+	Mytomycin-C	Selective CSC toxicity	Phase II trial	(177)
Prostate Cancer	Prostatic epithelial stem cells	CD44+, TROP2+	Androgen deprivation therapy	Targets CSC signaling pathway	Standard of care for advanced prostate cancer	(178, 179)
Kidney Cancer	Renal progenitor cells	CD105+, CD133+	VEGF inhibitors	Anti-angiogenic against CSCs	Phase III trial of bevacizumab + IFN-α	(180, 181)
Acute Myeloid Leukemia	Hematopoietic stem cells	CD34+, CD38-	DOT1L inhibitors	Disrupts CSC epigenetic regulation	Phase 1b/II trial of pinometostat	(182, 183)

### Monoclonal and bispecific antibodies

Antibodies targeting surface antigens and pathways selectively enriched in CSCs based on cell-of-origin are another promising approach. Glembatumumab vedotin targets glycoprotein NMB overexpressed on CSCs across cancer types including breast cancers of basal/myoepithelial origin (184). Additionally, antibodies against the epithelial cell adhesion molecule (EpCAM) preferentially expressed on liver CSCs derived from hepatic progenitor cells, and anti-CD47 antibodies blocking “don’t eat me” signaling in leukemia CSCs arising from hematopoietic stem cells have entered clinical testing (185, 186). Ongoing research aims to identify novel cell-origin-associated CSC antigens amenable to antibody targeting.

### Gene therapies

Leveraging knowledge of genetic drivers and dependencies based on cell-of-origin offers opportunities for gene therapy. Delivering mutant KRAS-targeted CRISPR constructs preferentially suppresses acinar cell-derived pancreatic CSCs exhibiting aberrant KRAS activity (187). Additionally, suicide gene strategies using a herpes simplex virus thymidine kinase transgene and ganciclovir prodrug show promise against glioblastoma CSCs derived from neural stem cells (188). Enhancing selective targeting of gene therapies against cell-origin-defined CSC populations is warranted.

### Combination strategies

**Figure 2:**
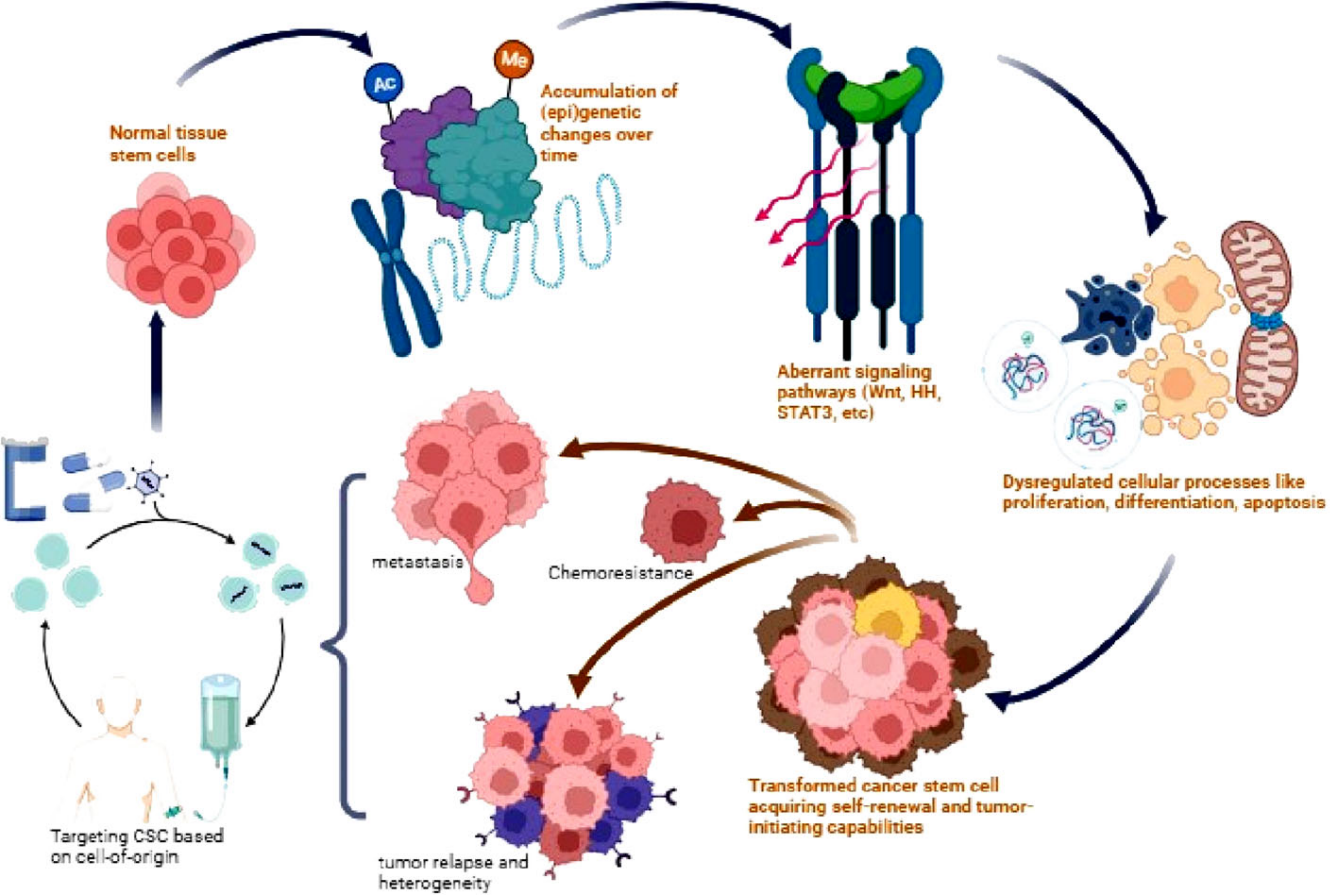
Origin and therapeutic targeting of CSCs. Normal tissue stem cells accumulate genetic and epigenetic alterations over time leading to dysregulation of signaling pathways such as Wnt, Hedgehog (HH), STAT3, and AKT. This results in aberrant stem cell processes and the transformation into CSCs with acquired self-renewal and tumor-initiating capabilities. CSCs drive tumor heterogeneity, metastasis, chemotherapy resistance, and relapse. Targeting dysregulated CSC signaling pathways provides a potential therapeutic approach to eliminate this tumorigenic population and improve patient outcomes.

Given the marked heterogeneity of CSCs, concurrently targeting bulk tumor cells and CSC subpopulations dependent on their unique cell-of-origin are under active evaluation. For example, simultaneously targeting HER2 and EGFR or PIK3CA signaling using trastuzumab and lapatinib in HER2+ progenitor-derived luminal breast CSCs shows synergistic activity (189, 190). Combined inhibition of MEK and Bcl-2 selectively suppresses intestinal crypt stem cell-derived colorectal CSCs exhibiting co-activation of MAPK and anti-apoptotic pathways (191). Moving forward, high-dimensional mapping of cell-origin CSC vulnerabilities using single-cell omics promises to inform rational combination therapies (192). Combining personalized and multi-modal approaches holds great promise for achieving long-term remissions and overcoming therapy resistance, which requires extensive research efforts focused on precision targeting of heterogeneous and adaptable CSC populations (**Figure 2**).

## Precision targeting of chemoresistant cancer stem cells in solid tumors based on cell-of-origin

Eradicating chemoresistant CSCs is critical to improve outcomes in solid tumors. This section discusses emerging strategies against cell-origin-defined CSCs across diverse cancer types.

### Breast cancer

In triple negative breast cancer (TNBC), CSCs arise from basal/myoepithelial progenitors and are marked by ALDH+ and CD44+/CD24- (193). Overexpression of ALDH1A3 isoform in these CSCs contributes to chemoresistance. Accordingly, the ALDH inhibitor disulfiram preferentially inhibits ALDH1A3 activity, reduces CSC populations, and re-sensitizes TNBC models to taxanes (139). Additionally, oncolytic viruses like MG1MAGEA3 specifically target MAGE-A3 expressed on ALDH+ TNBC CSCs, diminishing these cells and enhancing chemotherapy efficacy (140).

### Colorectal cancer

Colorectal CSCs marked by Lgr5 and CD44 arise from intestinal crypt base stem cells and drive chemoresistance *via* Wnt pathway activation (53). Porcupine inhibitor LGK974 suppresses Wnt signaling specifically in Lgr5+ CSCs, potentiating oxaliplatin therapy (141). Napabucasin inhibits STAT3-NANOG signaling preferentially activated in Lgr5+/CD44+ CSCs, reducing this subset and sensitizing to irinotecan (12, 142).

### Glioblastoma

Glioblastoma CSCs expressing CD133 and Nestin originate from neural stem cells and utilize aberrant Hedgehog pathway signaling (144). Hedgehog inhibitor GDC-0449 selectively antagonizes SHH ligands in Nestin+ CSCs, decreasing these cells, enhancing chemoradiation response, and delaying recurrence (144). STAT3 inhibitor WP1066 suppresses stemness transcription factors like NANOG in CD133+ CSCs, improving temozolomide efficacy (143).

### Cervical cancer

Cervical CSCs marked by ALDH1A1 and CD133 arise from transformed cervical epithelial progenitors and upregulate oxidative stress response pathways (194). Salinomycin selectively inhibits stress response enzymes highly expressed in CD133+/CD44+ CSCs, reducing this subset and re-sensitizing tumors to docetaxel (145).

### Ovarian cancer

Ovarian CSCs expressing ALDH1 and CD117 originate from transformed fallopian tube epithelia and exhibit platinum resistance. All-trans retinoic acid (ATRA) binds retinoic acid receptors on ALDH1A1+/CD117+ CSCs, inducing differentiation and re-sensitizing tumors to platinum therapy (147). ALDH inhibitor disulfiram decreases ALDH1A1+ CSCs, abrogating taxane resistance (146).

### Lung cancer

Lung CSCs marked by CD133 and ALDH derive from transformed lung epithelial cells and drive platinum resistance (195). ATRA induces differentiation of ALDH+ CSCs, reducing these cells and sensitizing tumors to cisplatin (160). Oncolytic adenovirus ZD55-TRAIL targets SOX2^high^NANOG^high^MRP1^high^ lung CSCs *via* CAR/integrin receptors, diminishing these cells and augments doxorubicin, vinblastine, cisplatin, and 5-FU efficacy (158).

### Hepatocellular carcinoma

Hepatocellular carcinoma CSCs marked by CD90 originate from hepatic progenitor cells and exhibit sorafenib resistance mediated by H3K9 methylation and stemness genes (196). Histone methyltransferase inhibitor GSK126 suppresses aberrant H3K9 methylation in CD90+/EZH2+ CSCs, impairing stemness and sensitizing to sorafenib (161, 196).

### Pancreatic cancer

Pancreatic CSCs expressing CD44 and ESA derive from transformed pancreatic progenitors and utilize hedgehog signaling to maintain stemness and confer gemcitabine resistance (148). Hedgehog inhibitor LDE225 blocks SMO-mediated hedgehog pathway activation specifically in CD44+/ESA+ CSCs, diminishing these cells and sensitizing tumors to gemcitabine (148, 149).

### Neuroblastoma

Neuroblastoma CSCs marked by CD114 and ALK originate from neural crest progenitors and exhibit chemotherapy resistance (197). ALK and mTOR inhibitors like crizotinib and everolimus preferentially target ALK and mTOR-driven survival pathways upregulated in ALK+ and CD114+ CSCs respectively, re-sensitizing tumors to chemotherapy (150, 151).

### Bladder cancer

Bladder CSCs enriched in CD44 arise from transformed basal urothelial progenitors and drive cisplatin resistance *via* NF-κB, Wnt and Notch signaling (152). Curcumin analog EF24 potently inhibits these pathways preferentially activated in CD44+ CSCs, reducing these cells and re-sensitizing tumors to cisplatin (153).

### Prostate cancer

ALDH1A1+ castration-resistant prostate CSCs originate from transformed basal epithelial progenitors and exhibit aberrant STAT3/AKT signaling (154). Niclosamide suppresses STAT3 and AKT signaling specifically in ALDH1A1+ CSCs, augmenting tumor response to androgen deprivation (154).

### Kidney cancer

CD105+ Kidney CSCs derive from nephron progenitors in clear cell renal carcinoma and confer radioresistance *via* mTOR and Chk1 signaling (198). The combination of mTOR and Chk1 inhibition, demonstrating cytotoxic and anti-proliferative effects, with potential translational value for treatment, has been shown to effectively target oncogenic pathways in CD105+ renal CSCs, leading to increased radiosensitivity in ccRCC (155–157). Additionally, the inhibition of PLK1, a cell-cycle-related kinase, has been identified as a potential therapeutic target in clear cell renal cell carcinoma, further supporting the potential of combined mTOR and Chk1 inhibition in this context (199).

Despite advances in CSC-targeting therapies based on cell-of-origin properties, several challenges remain. The intratumoral and intertumoral heterogeneity of CSCs presents significant challenge to identifying universal CSC-specific targets, thus, identifying robust and universal cell-origin-based CSC-specific markers remains an ongoing area of research (**Tables 1**, **2**). The dynamic interplay between CSCs and the TME further complicates therapy responses and necessitates consideration. Preclinical models that accurately depict the cell-of-origin context and TME are essential for evaluating therapeutic interventions (**Table 1**). Additionally, Biomarker discovery and patient stratification based on cell-of-origin characteristics are critical for implementing personalized CSC-targeting therapies in clinical settings (**Table 2**). Overcoming these challenges requires integrative advanced omics technologies, preclinical models that mimic the TME, and well-designed clinical trials.

**Figure 3:**
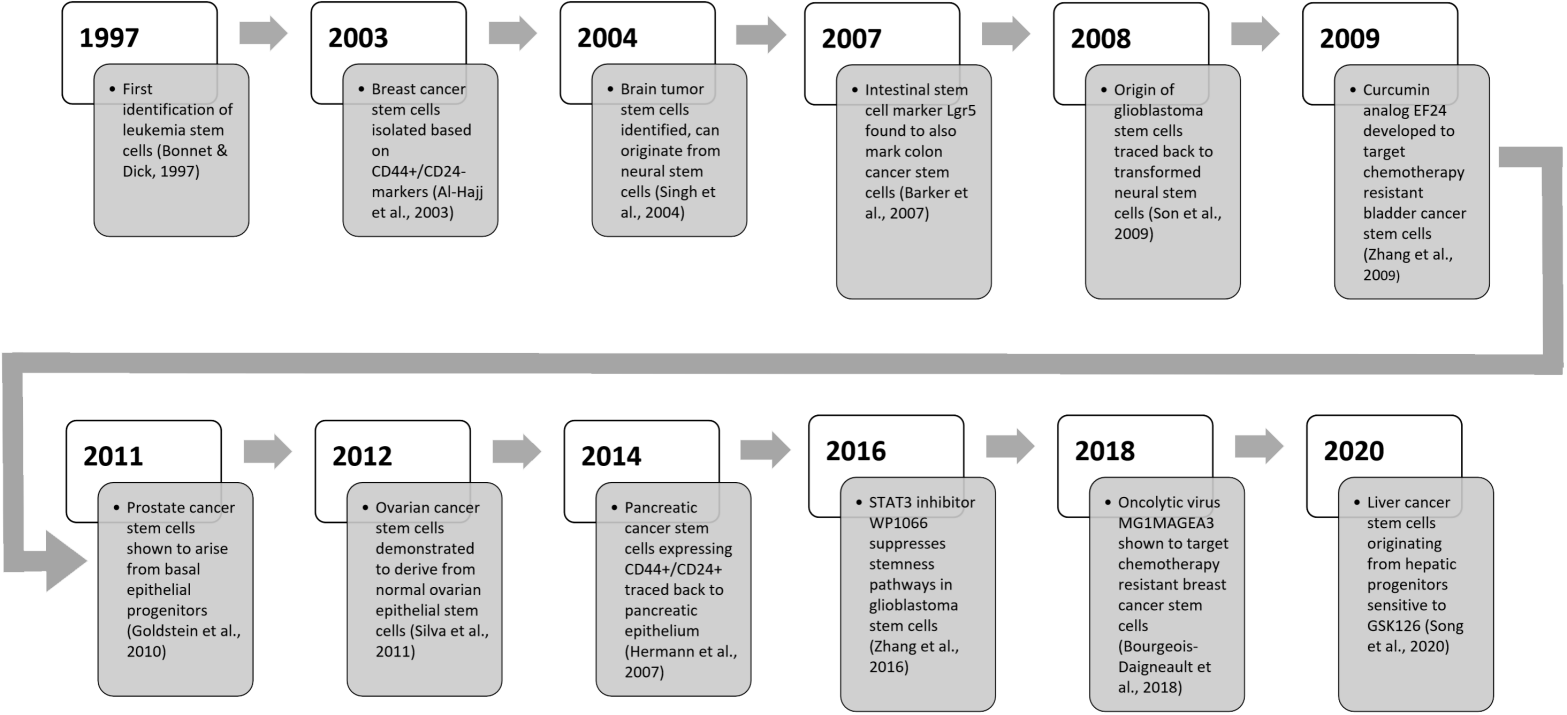
An updated timeline of key discoveries related to cancer stem cell research.

Personalized and multi-modal cancer treatments using small-molecule inhibitors, monoclonal antibodies, gene therapies, and combination approaches offer promising avenues for improving cancer treatment outcomes. Continued research on the complex interactions between CSCs and their cell-of-origin is essential for developing transformative anticancer therapeutic strategies and achieving lasting remissions (**Figure 3**). Overall, despite lingering challenges including tumor heterogeneity, dynamic CSC plasticity, and resistance mechanisms, unraveling cell-origin allows tailored targeting of chemoresistant CSC subsets across diverse solid tumors.

## Critical appraisal and controversy surrounding cancer stem cells, cells-of-origin, and their clinical/therapeutic utility

The concepts of CSCs and cell-of-origin have transformed and advanced our understanding of tumor biology, but their intricacies have also sparked debate and exposed critical gaps in knowledge (14). CSCs are characterized by their ability to self-renew and recreate the full heterogeneity of the original tumor, but isolating pure CSC populations and identifying immutable CSC markers has proven extremely challenging (200). Several candidate CSC markers have been proposed, but demonstrating specificity and reproducibility has been difficult.

Against initial propositions that CSCs are exceptionally rare within tumors, accounting for <5% of cells, mounting evidence indicate non-CSC differentiated tumor cells can readily de-differentiate and acquire stem-like behavior, questioning the rarity of CSCs (201). This plasticity allows non-CSCs convert into CSCs, especially in response to therapies targeting the differentiated tumor bulk. Furthermore, CSCs exhibit inherent plasticity, dynamically transitioning between stem-like and non-stem cell states, enabling CSC flexibility and adaptability which consequently complicates therapeutic efforts to permanently eliminate them (20). In summary, CSCs demonstrate plasticity, context-dependency, and resistance to rigid definitions, creating complex challenges in isolating, studying, and targeting these shifting cells.

The cell-of-origin concept refers to the normal cell type, whether a stem cell, progenitor cell, or mature differentiated cell, that initially acquires the first cancer-causing mutations and undergoes transformation to give rise to full malignancy. However, it is difficult to definitively determine the cell-of-origin retrospectively due to limitations and challenges (9). After a tumor has developed, reconstructing the initiating events to pinpoint the cell-of-origin is difficult, if not impossible, in many cases. Moreover, different potential cells-of-origin, including progenitor or stem cells, can ultimately converge on very similar tumor phenotypes after accumulating mutations. Emerging evidence indicates that combined mutations occurring simultaneously across multiple cell types within a tissue may be required for full malignant transformation, rather than mutations within a single discrete cell (202), thus, obscuring efforts to clearly delineate the initiating cell-of-origin.

The goal of identifying the cell-of-origin is to develop therapies that specifically target malignant stem cells while sparing normal stem cells. However, developing therapies that discriminate between malignant and normal stem cells remains difficult. For example, despite preclinical data showing DOT1L inhibitor pinometostat exhibits10-fold higher toxicity against leukemia stem cells than normal hematopoietic stem cells, nearly 50% of acute myeloid leukemia patients relapsed after DOT1L inhibition, highlighting the challenges of translating cell-of-origin insights into clinical practice (182, 203).

Clinically, emerging therapies directly targeting CSCs, such as STAT3 inhibitor napabucasin and ABL inhibitor asciminib, have shown initial efficacy in early phase trials, offering proof-of-concept for CSC-directed approaches (204). However, resistance and relapse continue to pose substantial challenges. In studies of ALDH inhibitors, suppression of CSC populations was only transient before their reconstitution, highlighting the need for combination treatments (205). However, directly linking patient clinical response to the specific effects on CSCs is complicated by the realities of intratumor heterogeneity and CSC plasticity (5). CSCs can dynamically transition between cell states and repopulate the CSC pool, limiting durable responses to single agent CSC-targeting drugs.

The rarity of CSCs poses inherent difficulties for analysis and evaluation, especially in the context of tumor heterogeneity. The low CSC frequency makes obtaining sufficient material for in-depth profiling challenging. Furthermore, low initial CSC frequency can cloud clinical response assessments if the resolution is inadequate, since even small surviving CSCs can re-initiate tumor growth (206). This has stirred debate on whether rarity *versus* abundance is more therapeutically disadvantageous. Thus, despite promising preliminary results, realizing the full potential of CSC-targeted therapies will require overcoming hurdles related to tumor evolution, plasticity, heterogeneity, and rarity through rational combination strategies and high-resolution monitoring.

The paradigm-shifting concept of CSCs and cell-of-origin holds immense promise for transforming cancer prevention, treatment, and overcoming therapeutic resistance. However, capitalizing on their full potential requires navigating tremendous knowledge gaps and inherent complexities. A key challenge is prospectively isolating definitive purified CSC subsets for characterization because of biomarker ambiguity and interconversions between states (11, 14). Preventing resistance requires innovative, adaptive therapies blocking CSC evolution and plasticity (59). Selectively eliminating CSCs while sparing normal stem cells remains an ongoing quest requiring deeper appreciation of stemness vulnerabilities (182). Realizing clinical advances necessitates embracing CSC complexity through emerging tools like high-dimensional single-cell profiling, organoids, and computational modeling that capture dynamic stem cell ecosystem interactions (207, 208). By unraveling the intricacies of malignant stem cell biology, pioneering interdisciplinary science can transform cancer into a durable chronic condition rather than a deadly disease.

## Challenges and future directions

Despite advances in our understanding of the interplay between CSCs and the cell-of-origin, several challenges persist. First, cell-origin-based identification and characterization of CSCs remains challenging due to CSC and TME heterogeneity. Improved single-cell sequencing technologies coupled with innovative lineage tracing approaches may provide deeper insights into distinct cellular origins of CSCs within heterogeneous tumors (201, 209). Second, CSC plasticity complicates their targeting because of their dynamic transitions between stem-like and differentiated states. Improved understanding of the epigenetic and signaling mechanisms regulating CSC plasticity could lead to novel strategies to maintain CSCs in a more differentiated state, susceptible to conventional therapies (210, 211). Third, TME plays a critical role in regulating CSC behavior and therapeutic responses. Interactions with immune cells, fibroblasts, and the ECM contribute to CSC maintenance and therapy resistance (5, 212). Investigating the crosstalk between CSCs and TME may unveil new therapeutic targets and combination strategies to overcome therapy resistance. Fourth, the development of reliable CSC-specific biomarkers based on cell-of-origin properties is essential for clinical translation. Biomarkers that can accurately identify and isolate CSCs from patient samples would facilitate the design of targeted therapies and monitoring of treatment responses (213). Continued efforts in biomarker discovery and validation are crucial for advancing CSC-targeted therapies.

Furthermore, integrated multi-omics approaches are required to fully characterize and map the cell-of-origin-specific molecular vulnerabilities and dependencies of CSCs. Additionally, novel *ex vivo* and *in vivo* models that more accurately recapitulate the complex native TME are required to better understand the extrinsic regulation of CSC behavior by the surrounding niche. Standardization of protocols for isolating and validating CSCs across research groups would allow for improved comparisons and reproducibility between studies. As evident in **Table 2**, clinical trials evaluating cell-of-origin-guided therapies and combination approaches thatspecifically target CSCs are critical for translating research findings to benefit patients (162-159). Finally, the development and validation of reliable biomarkers and assays to track and profile CSCs longitudinally in patients could enable earlier detection of minimal residual disease and facilitate more tailored, adaptive treatment monitoring approaches.

## Conclusion

The intricate relationship between CSCs and their cell-of-origin is an exciting frontier in cancer research. Understanding this interaction provides insights into the molecular basis of tumor initiation, therapeutic resistance, and patient outcomes. The path forward demands interdisciplinary collaboration to unlock transformative insights that propel more effective, adaptive, and durable therapies targeting the self-renewing core of tumors; ultimately making curative outcomes feasible and fundamentally altering the prognosis of cancer patients worldwide.

## Author contributions

OB: Conceptualization, Data curation, Formal analysis, Investigation, Methodology, Project administration, Resources, Supervision, Validation, Visualization, Writing – original draft, Writing – review & editing. C-CC: Formal analysis, Funding acquisition, Methodology, Writing – original draft. TP: Formal analysis, Methodology, Writing – review & editing, Supervision. AW: Formal analysis, Methodology, Project administration, Writing – review & editing.
